# A novel mRNA vaccine, TGGT1_278620 mRNA-LNP, prolongs the survival time in BALB/c mice with acute toxoplasmosis

**DOI:** 10.1128/spectrum.02866-23

**Published:** 2023-12-01

**Authors:** Yizhuo Zhang, Shiyu Li, Hongkun Chu, Jing Li, Shaohong Lu, Bin Zheng

**Affiliations:** 1 Laboratory of Pathogen Biology, School of Basic Medicine and Forensics, Hangzhou Medical College, Hangzhou, China; 2 School of Basic Medicine and Forensics, Hangzhou Medical College, Hangzhou, China; 3 Engineering Research Center of Novel Vaccine of Zhejiang Province, Hangzhou Medical College, Hangzhou, China; 4 Key Laboratory of Bio-Tech Vaccine of Zhejiang Province, Hangzhou Medical College, Hangzhou, China; Clemson University, Clemson, South Carolina, USA

**Keywords:** *Toxoplasma gondii*, vaccine, mRNA, lipid nanoparticle, immune response

## Abstract

Toxoplasmosis is a global health issue; however, there is a lack of safe and effective drugs and vaccines. Herein, we developed a novel mRNA vaccine, TGGT1_278620 mRNA-lipid nanoparticle (LNP), and evaluated its safety and efficacy in BALB/c mice. The vaccine elicited a significant increase not only immunoglobulin (Ig)G and IgG subclasses (IgG2a and IgG1) antibodies but also interleukin (IL)-12, interferon-gamma, IL-4, and IL-10 cytokines. We showed that specific cellular immunity and humoral immunity were activated by combining splenocyte proliferation, cytotoxic T-cell activity, and T-cell surface molecule CD8 and CD4 ratios. Dendritic cell signaling pathway members interferon regulatory factor 8, T-Box 21, and nuclear factor kappa B were analyzed at the mRNA and protein levels, and increased differentiation and maturation were observed by examining dendritic cell surface molecules (CD83, CD86, MHC-I, and MHC-II) using flow cytometry. Finally, the survival time of the vaccinated mice was significantly prolonged after *Toxoplasma gondii* RH challenge, and the adoptive transfer of splenocytes and sera from the vaccinated mice also significantly prolonged survival. These findings suggested that the TGGT1_278620 mRNA-LNP vaccine might be a promising candidate for further development in anti-toxoplasmosis therapy.

**IMPORTANCE:**

*Toxoplasma gondii*, an obligate intracellular eukaryotic parasite, can infect about one-third of the world’s population. One vaccine, Toxovax, has been developed and licensed commercially; however, it is only used in the sheep industry to reduce the losses caused by congenital toxoplasmosis. Various other vaccine approaches have been explored, including excretory secretion antigen vaccines, subunit vaccines, epitope vaccines, and DNA vaccines. However, current research has not yet developed a safe and effective vaccine for *T. gondii*. Here, we generated an mRNA vaccine candidate against *T. gondii*. We investigated the efficacy of vaccination with a novel identified candidate, TGGT1_278620, in a mouse infection model. We screened *T. gondii*-derived protective antigens at the genome-wide level, combined them with mRNA-lipid nanoparticle vaccine technology against *T. gondii*, and investigated immune-related factors and mechanisms. Our findings might contribute to developing vaccines for immunizing humans and animals against *T. gondii*.

## INTRODUCTION

*Toxoplasma gondii*, an obligate intracellular eukaryotic parasite belonging to the phylum Apicomplexa, can infect a significant portion of the global population (1). Infection rates in certain regions, such as Central America, South America, and continental Europe, range from 30% to 90%. Toxoplasmosis is a leading illness associated with foodborne hospitalizations and deaths, ranking as the second leading cause of death among foodborne illnesses in the USA (2). The main sources of foodborne transmission in humans are undercooked meat, particularly pork, lamb, and game, and soil contaminated with cat feces on raw fruit and vegetables.

While healthy individuals with normal immunity might not experience clinical symptoms, immunocompromised individuals, especially those with HIV/AIDS or who have undergone organ transplants, are at risk of severe toxoplasmosis (3, 4). Pregnant women are also more susceptible, and the infection can lead to severe consequences for the fetus (5). Current treatment options for toxoplasmosis, such as the combination of pyrimethamine and sulfadiazine, target the active stage of the infection but have significant failure rates. These treatments often require extended courses, have potential side effects, such as bone marrow suppression, and exhibit significant toxicity (6). *T. gondii* infection is common in warm-blooded animals and constantly threatens humans and livestock. Eradication of *T. gondii* infection is almost impossible because of the wide range of natural reservoirs. For *T. gondii* infection, a vaccine would be a complement to treatment. In addition, a vaccine targeting high-risk groups might be valuable for women of childbearing age or HIV-positive patients. Consequently, developing an effective vaccine remains the preferred approach for prevention.

Only one vaccine, Toxovax (MSD, New Zealand), has been developed and licensed commercially (7). Toxovax is a live attenuated vaccine used only in the sheep industry to reduce losses caused by congenital toxoplasmosis. Various other vaccine approaches have been explored, including excretory secretion antigen (ESA) vaccines, subunit vaccines, epitope vaccines, exosome vaccines, DNA vaccines, and mRNA vaccines. However, no commercially available vaccines have been marketed (8, 9). While ESA immunization does not offer complete immune protection and struggles to overcome the complex strains and life cycle of *T. gondii*, subunit vaccines provide limited immune protection and often require carrier or adjuvant delivery. Exosome vaccines suffer from quality control issues, inconsistent purification standards, and insufficient manufacturing, and DNA vaccines have been extensively studied but have not been used in humans (10).

In contrast, mRNA vaccines have already been licensed and used clinically (e.g., the COVID-19 mRNA vaccine) (11). mRNA vaccines offer significant advantages, including a shorter design and testing timeline and an effective and long-lasting immune response. They have a significant advantage against outbreaks (12, 13). Given the rapid progress of mRNA vaccines, especially in immune informatics and *in vivo* delivery methods, the prospects for producing mRNA vaccines at low cost and high quality in veterinary medicine are very bright.

The objective of the present study was to determine the immunogenic activity of TGGT1_278620 (TG_620), which was screened by prediction of *T. gondii* epitopes. The antibody level, cytokines, lymphocyte proliferation, and cytotoxic T lymphocyte (CTL) effects were used to evaluate the efficacy of the constructed TG_620 mRNA-lipid nanoparticle (LNP) vaccine. Finally, the immune protection of the vaccine was evaluated by *T. gondii* RH strain challenge and adoptive transfer. The TG_620 mRNA-LNP induced high levels of specific antibodies [immunoglobulin (Ig)G] and cytokines [interferon-gamma (IFN-γ), interleukin (IL)-12, IL-10, and IL-4] in mice. It induced specific cellular and humoral immune responses and significantly prolonged the survival time of mice challenged with *T. gondii*. These findings suggested that the TG_620 mRNA-LNP vaccine might be a promising candidate for further development in anti-toxoplasmosis research.

## RESULTS

### TG_620 was identified to have better B- and T-cell epitopes scores than SAG1

**Fig 1:**
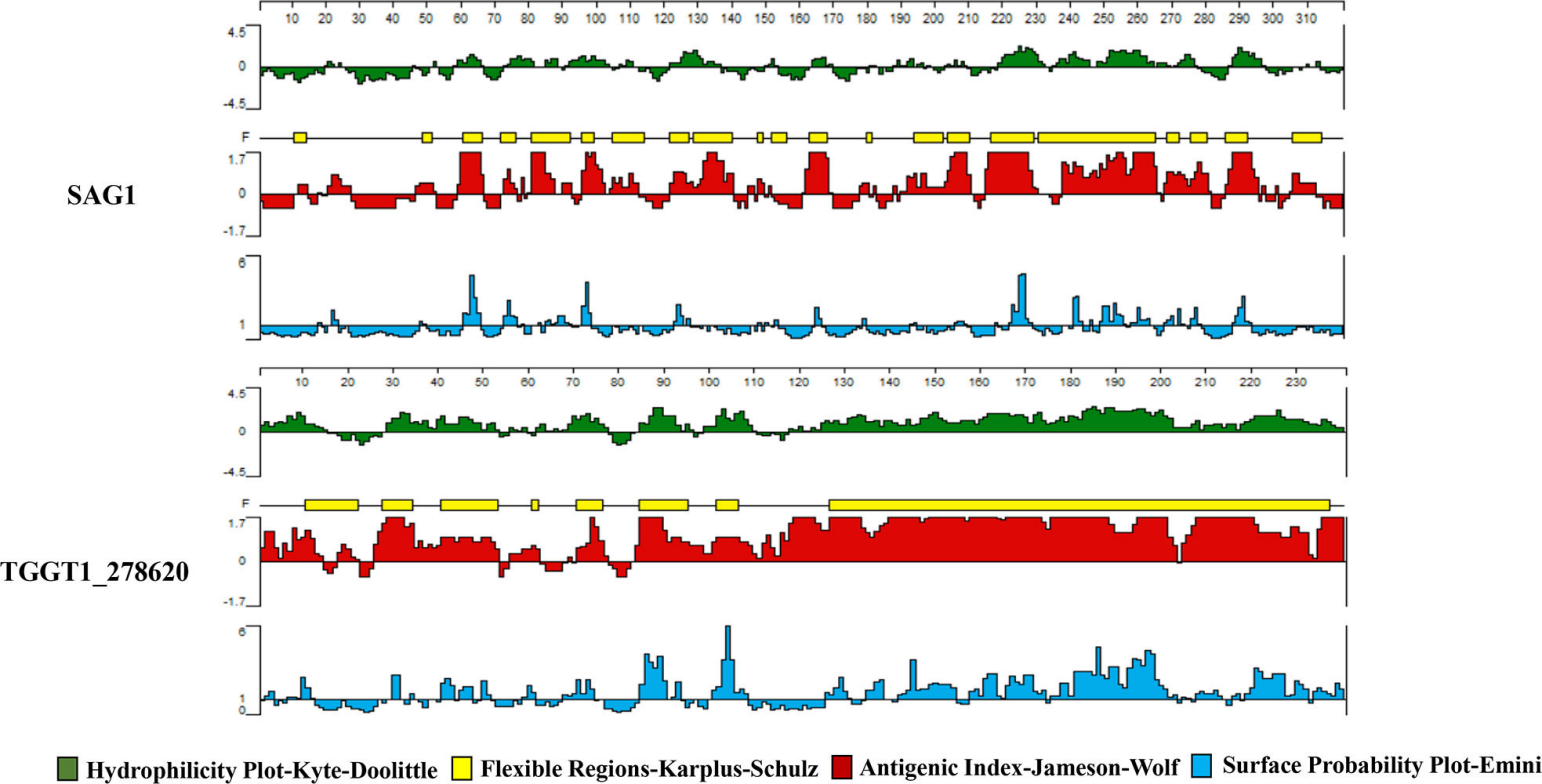
The plot of the DNASTAR-predicted hydrophilicity, flexible regions, antigenic index, and surface probability of the linear B-cell epitopes of TG_620 compared with those of SAG1.

Numerous antigens have been identified as potential vaccine candidates against *T. gondii*, such as surface antigen proteins (TgSAGs), rhoptry protein (TgROPs), microneme proteins (TgMICs), and others (14). Despite choosing diverse immunization routes, animal models, doses, and vaccine production processes, none of the vaccines developed based on these candidates have demonstrated complete protection against *T. gondii*. Secreted proteins play a crucial role in mediating the interaction between hosts and pathogens (15); therefore, this study focused on analyzing *T. gondii*-secreted proteins at the genome-wide level to find possible vaccine candidates. Using the *T. gondii* database (ToxoDB), we first screened for secreted proteins containing both a transmembrane domain and a signal peptide at the whole-genome level. Of over 8,000 genes in the *T. gondii* genome, only 233 had transmembrane domains and signal peptides. A vaccine candidate with a high potential for success is often identified by a bioinformatic analysis that predicts a high T-/B-cell epitope score (16, 17). Based on published data, *T. gondii* surface antigen 1 (SAG1) has strong immunogenicity and immunoprotective effects and is considered a promising candidate antigen and a major target for inducing host immune responses (18). Therefore, SAG1 was chosen as the reference protein for this experiment. We used DNASTAR (PROTEAN program) and the Immune Epitope Database (IEDB) to perform T-/B-cell epitope analysis on the screened 233 genes and compared them with SAG1. We obtained several candidate proteins, among which TGGT1_278620 protein (TG_620) showed the best T-/B-cell epitope prediction scores. This study used the DNASTAR software to predict hydrophilicity, flexible regions, antigenic index, and surface probability of TG_620. Figure 1 shows that most regions of TG_620 protein exhibited hydrophilicity and flexibility, and the protein had ideal surface probability and antigenic index.

To analyze T helper (Th)-cell epitopes, IEDB was used. The database predicted the half maximal inhibitory concentration (IC_50_) values of peptides from TG_620 and SAG1 proteins when binding to the major histocompatibility complex (MHC) class II molecule. Table 1 shows the minimum percentile ranks of each MHC-II allele on TG_620. The IC_50_ values of H2-IAb, H2-IAd, and HLA-DRB1*01:01 from TG_620 were lower than those from SAG1, indicating that TG_620 protein is likely to have strong binding to MHC-II molecules.

**TABLE 1:** qRT-PCR primers used to amplify the *p65*, *Tbet*, *Irf8*, and *Actb* (β-actin) genes designed by DNASTAR software

Primer name	Sequence
NF-κB p65-F	5′-GAACCAGGGTGTGTCCATGT-3′
NF-κB p65-R	5′-TCCGCAATGGAGGAGAAGTC-3′
T-bet-F	5′-GCCAGGGAACCGCTTATATG-3′
T-bet-R	5′-TGGAGAGACTGCAGGACGAT-3′
IRF8-F	5′-GCTGATCAAGGAACCTTGTG-3′
IRF8-R	5′-CAGGCCTGCACTGGGCTG-3′
β-Actin-F	5′-GCTTCTAGGCGGACTGTTAC-3′
β-Actin-R	5′-CCATGCCAATGTTGTCTCTT-3′

The bioinformatic analysis showed that TG_620 protein had better linear B-cell epitope scores than SAG1 and was likely to bind to MHC-II strongly.

### TG_620 mRNA construct was successfully expressed in 293T cells and C2C12 cells with high LNP EE

**Fig 2:**
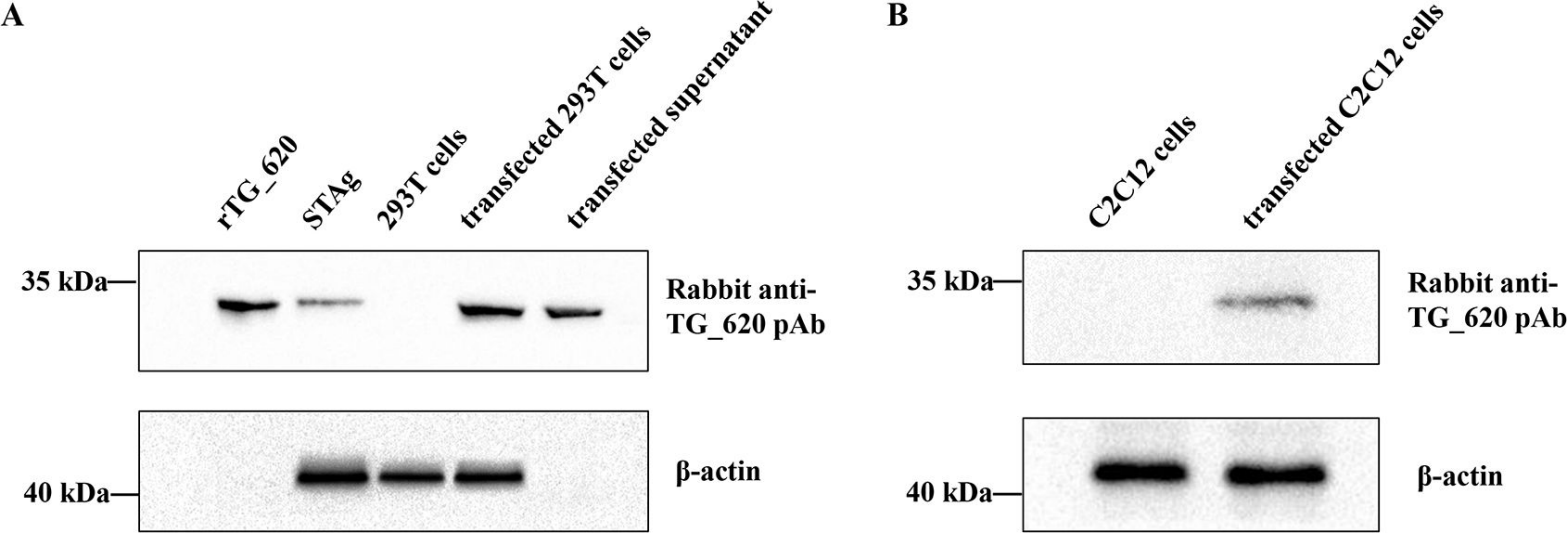
Expression of TG_620 mRNA in 293T cells and TG_620 mRNA-LNP in C2C12 cells. (**A**) WB detection of the expression of TG_620 in rTG_620, STAg, 293T cell lysates, and the supernatant of transfected 293T cells. rTG_620: the recombinant pET28a-TG_620; STAg: soluble antigen of *T. gondii*; 293T cells: 293T cell lysates; transfected 293T cells: TG_620 mRNA transfected 293T cells; transfected supernatant: TG_620 mRNA transfected 293T cell supernatant. (**B**) WB detection of TG_620 mRNA-LNP in C2C12 cells. C2C12 cells: C2C12 cell lysates; transfected C2C12 cells: TG_200 mRNA-LNP transfected C2C12 cell lysates.

The TG_620 mRNA construct was transfected into 293T cells (human embryonic kidney cells), and the expression of the TG_620 protein was detected using western blotting (WB). As shown in Fig. 2A, we used a rabbit polyclonal antibody against TG_620 to detect the TG_620 protein *in vitro* (rTG_620), in soluble *T. gondii* antigen (STAg) preparations, in lysates of 293T cells, in lysates of 293T cells transfected with the TG_620 mRNA construct (transfected 293T), and in the supernatant of 293T cells transfected with the TG_620 mRNA construct (transfected supernatant). β-Actin was used as the internal control protein in this experiment. A single band in rTG_620 indicated the expression of rTG_620 *in vitro* and confirmed the specific binding of the rabbit polyclonal antibody against TG_620. A single band in STAg indicated that the TG_620 mRNA protein is a soluble antigen of *T. gondii*. The absence of a band in 293T cells but a single specific band in lysates of 293T cells transfected with the TG_620 mRNA construct indicated that TG_620 protein can be expressed in eukaryotic cells. In addition, a single band in the transfected supernatant indicated that TG_620 is secreted by 293T cells.

After ensuring the expression of TG_620 in eukaryotic cells, we transfected C2C12 cells (mouse leg muscle cells) with LNP-encapsulated TG_620 mRNA and detected the expression of TG_620 using WB, with β-actin as the internal control protein. In this study, muscle injection was chosen as the immunization route; therefore, it was important to ensure the expression of TG_620 in muscle cells. Therefore, C2C12 cells were selected for the transfection of TG_620 mRNA-LNP. As shown in Fig. 2B, the rabbit anti-TG_620 polyclonal antibody was used to detect TG_620 in the cell lysates of C2C12 cells and TG_620 mRNA-LNP-transfected C2C12 cells. No band was detected in C2C12 cells, but a single band was observed in the lysate of transfected C2C12 cells, indicating that the TG_620 protein could be expressed in C2C12 cells.

The encapsulation efficiency (EE) of TG_620 mRNA in the LNPs was examined by measuring free RNA concentrations and total RNA concentrations in the solution post-encapsulation. The results showed that the obtained EE was 96.56%.

### TG_620 mRNA-LNP induces higher levels of IgG antibodies and cytokines (IFN-γ, IL-12, IL-4, and IL-10)

**Fig 3:**
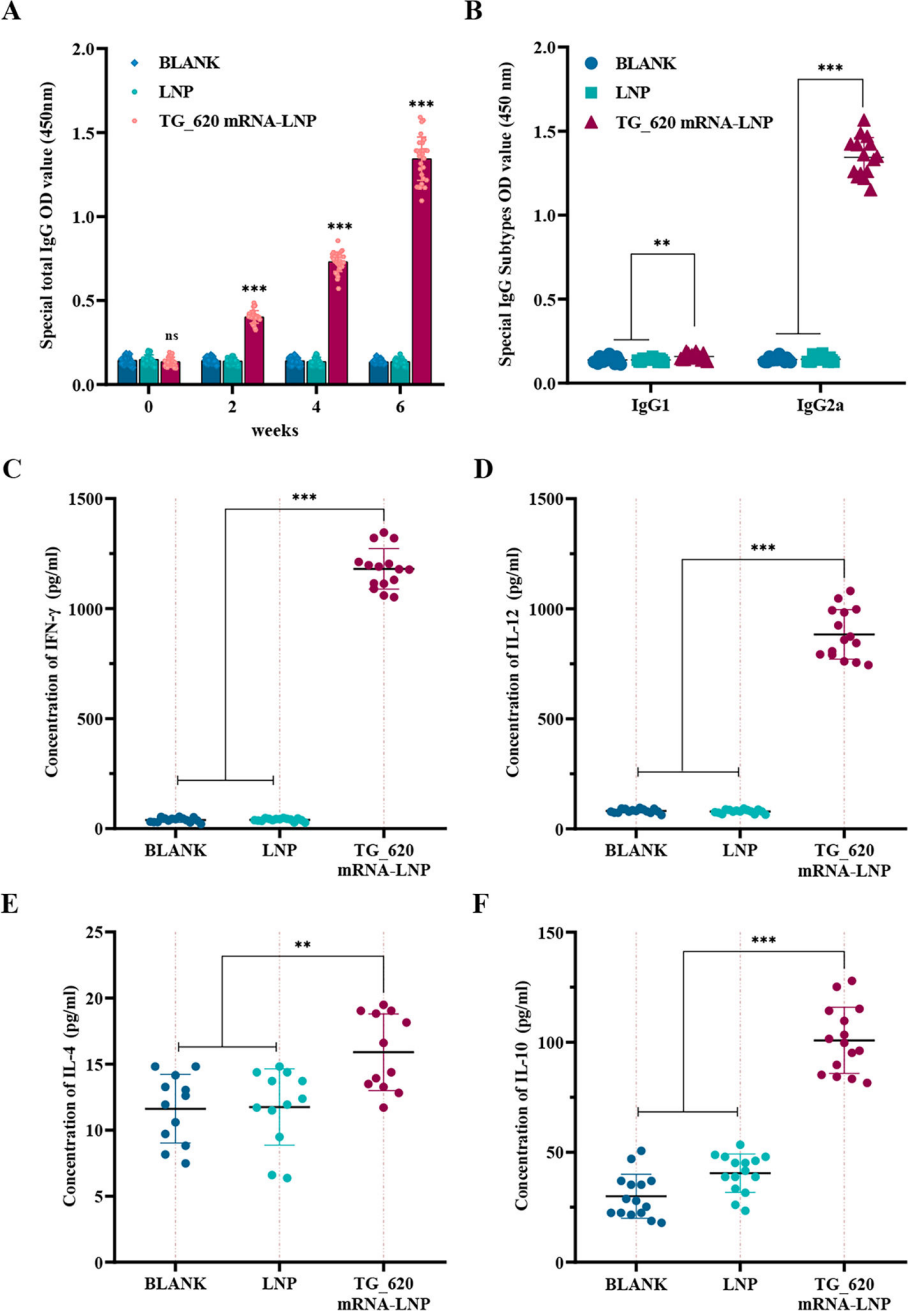
Detection of specific humoral immune responses and the cytokine production levels of splenocytes induced by TG_620 mRNA-LNP immunization. (**A**) Determination of IgG antibodies in BALB/c mice sera at 0, 2, 4, and 6 weeks after vaccination, *n* = 10/group. (**B**) Detection of IgG1 and IgG2a antibodies in mice immunized with TG_620 mRNA-LNP, LNP, or BLANK after the last immunization, *n* = 5/group. (**C**) The IFN-γ production level was significantly higher after immunization with TG_620 mRNA-LNP compared with that in the LNP and BLANK groups. (**D**) The IL-12 production level was significantly higher after immunization with TG_620 mRNA-LNP compared with that in the LNP and BLANK groups. (**E**) The IL-4 production level was significantly higher after immunization with TG_620 mRNA-LNP compared with that in the LNP and BLANK groups. (**F**) The IL-10 production level was significantly higher after immunization with TG_620 mRNA-LNP than in the LNP and BLANK groups. The horizontal axis is the cytokine concentration (pg/mL). *n* = 5/group. **P < 0.01 and ***P < 0.001. Data are shown as the means ± SD of three independent experiments.

To evaluate the *T. gondii*-specific antibody response, serum samples were collected from three groups of mice (BLANK, LNP, mRNA-LNP) and analyzed for total IgG and IgG subclasses (IgG1 and IgG2a) using an enzyme-linked immunosorbent assay (ELISA). As shown in Fig. 3A, the mRNA-LNP group showed an increasing trend in serum-specific IgG antibody levels with increasing immunization times. In the sixth week (after three immunizations), high levels of IgG were observed in the serum of the TG_620 mRNA-LNP-immunized groups (*P* < 0.001), while no significant difference was observed between the BLANK and LNP groups (*P* > 0.05). After the final immunization, the levels of specific IgG1 and IgG2a were measured in the serum of the three groups of mice. As shown in Fig. 3B, the levels of IgG1 and IgG2a were significantly higher in the TG_620 mRNA-LNP group than in the BLANK and LNP groups, while there was no significant difference in IgG1 and IgG2a levels between the BLANK and LNP groups. Additionally, the IgG2a/IgG1 ratio was higher in the mRNA-LNP group (8.49) than in the BLANK (1.02) and LNP groups (1.04).

IFN-γ and IL-12 play a crucial role in restricting the proliferation of tachyzoites during the early stages of *T. gondii* infection. IL-12 induces the release of IFN-γ and promotes the differentiation of Th1 cells, thereby controlling *T. gondii* infection. IL-10 and IL-4 responses can prevent an excessive inflammatory response by suppressing systemic Th1 cytokine production and inhibiting the development of immunopathology. Significant increases in cytokine levels (IFN-γ, IL-12, IL-4, and IL-10) were observed in the serum of mice immunized with TG_620 mRNA-LNP, with no significant difference observed between the BLANK and LNP groups. As shown in Fig. 3C, the concentration of IFN-γ was significantly higher (1,180.30 ± 23.82 pg/mL, *P* < 0.001) in mice immunized with TG_620 mRNA-LNP. As shown in Fig. 3D, the concentration of IL-12 was also significantly higher (883.80 ± 29.11 pg/mL, *P* < 0.001) in mice immunized with TG_620 mRNA-LNP. As shown in Fig. 3E and F, the levels of IL-4 and IL-10 were also significantly increased in TG_620 mRNA-LNP-immunized mice (IL-4: 15.90 ± 0.84 pg/mL, *P* < 0.01; IL-10: 100.80 ± 3.89 pg/mL, *P* < 0.001). All data are shown in Table 2.

**TABLE 2:** Cytokine production and the proliferative responses of splenocytes from BALB/c mice immunized with BLANK, LNP, or TG_620 mRNA-LNP group

Groups (*n* = 5)	Cytokine production (pg/mL)				Proliferation (SI)^*a*^
	IFN-γ	IL-12	IL-4	IL-10	
BLANK	39.52 ± 2.63	81.55 ± 2.35	11.62 ± 0.75	29.97 ± 2.59	0.17 ± 0.03
LNP	40.47 ± 1.65	79.12 ± 2.18	11.75 ± 0.83	40.45 ± 2.25	0.15 ± 0.04
TG_620 mRNA-LNP	1,180.31 ± 23.82^*b*^	883.80 ± 29.11^*b*^	15.90 ± 0.84^*b*^	100.80 ± 3.89^*b*^	2.62 ± 0.18^*b*^

^*a*^ SI, stimulation index.

^*b*^ *P* < 0.05 compared with the control groups.

These results indicated that a mixed Th1 and Th2 immune response was elicited in mice immunized with TG_620 mRNA-LNP, representing an effective immune response against *T. gondii* infection.

### Lymphocyte proliferation ability and CTL activity are enhanced in TG_620 mRNA-LNP-immunized mice

**Fig 4:**
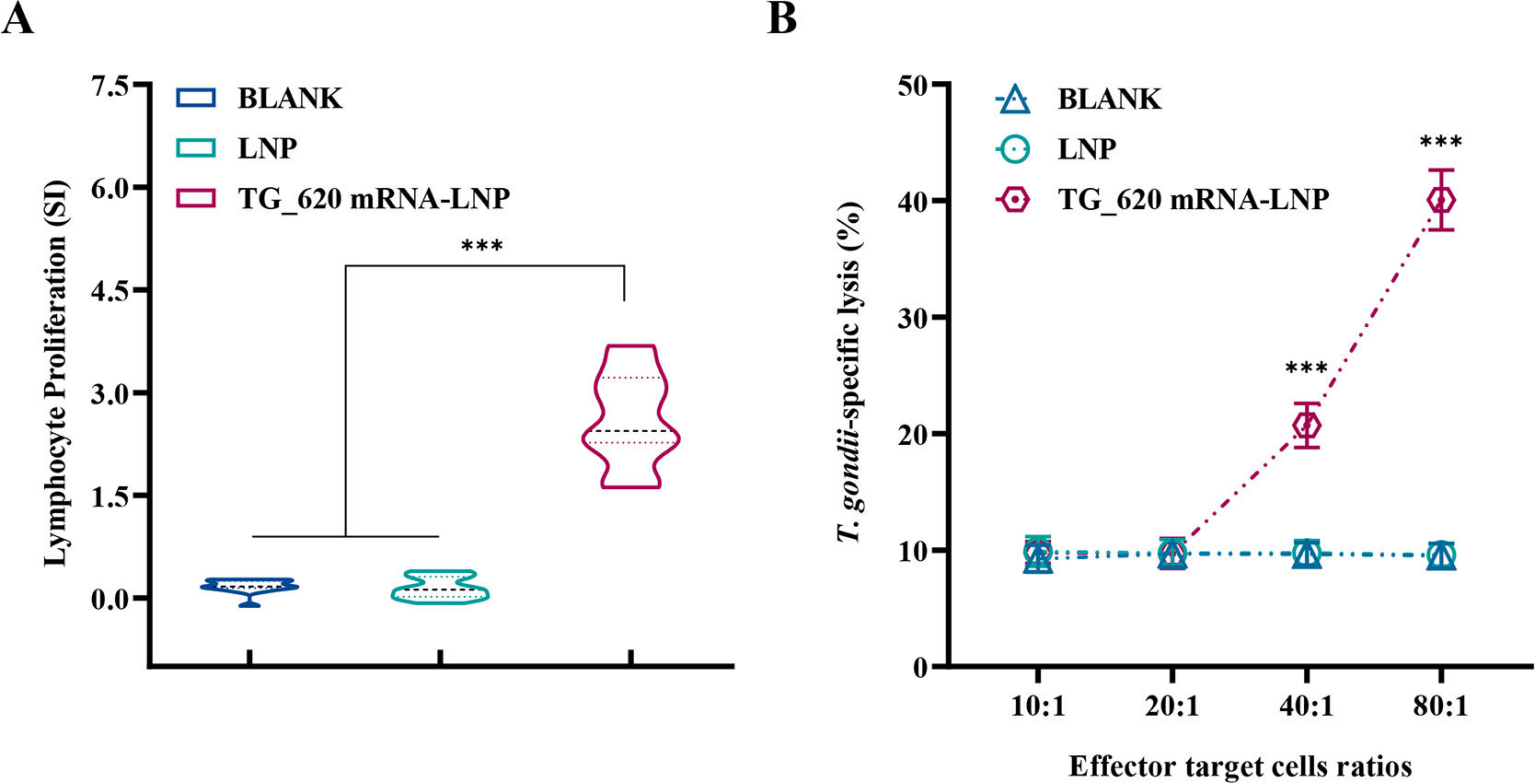
Splenocyte proliferation and CTL activities of splenocytes in TG_620 mRNA-LNP- and LNP-immunized or BLANK group mice. (**A**) Proliferative responses of splenocytes in BALB/c mice immunized with LNP, mRNA-LNP, or BLANK control, *n* = 5/group, ***P < 0.001. (**B**) CTL activities of splenocytes in BALB/c mice immunized with LNP, mRNA-LNP, or BLANK control. The vertical axis shows *T. gondii*-specific lysis as a percentage of the total possible lysis (%). The horizontal axis is the ratio of effector cells to target cells. *n* = 5/group, ***P < 0.001.

The proliferation and differentiation of lymphocytes are important in cellular immunity. In this experiment, we validated the specificity of the immune response induced by TG_620 mRNA-LNP vaccine using lymphocyte proliferation assays. Two weeks after the final immunization, splenocytes were isolated and purified from five mice in each group and used for lymphocyte proliferation assays. As shown in Fig. 4A, splenocytes from mice immunized with TG_620 mRNA-LNP showed a significantly higher proliferation activity than those from mice immunized with LNP and BLANK (*P* < 0.001). All data are shown in Table 2. There was no significant difference in lymphocyte proliferation ability between the LNP and BLANK groups. The significant increase in lymphocyte proliferation indicated the production of specific cellular immunity.

CTLs are one of the main effector cells of specific cellular immunity. Fig. 4B shows a significant difference when the effector-target cell ratio was 40:1 (*P* < 0.001). When the ratio was 80:1, the CTL activity reached higher values (*T. gondii*-specific lysis accounted for more than 40% of total possible lysis). There was no significant difference in splenic lymphocyte CTL activity between the LNP and BLANK groups (*P* > 0.05). These results showed that specific CTL responses were induced, and effective immune responses to intracellular pathogens were produced in TG_620 mRNA-LNP immunized mice.

### TG_620 mRNA-LNP could induce the expression of CD83, CD86, MHC-I, MHC-II, CD8, and CD4 cell surface molecules

**Fig 5:**
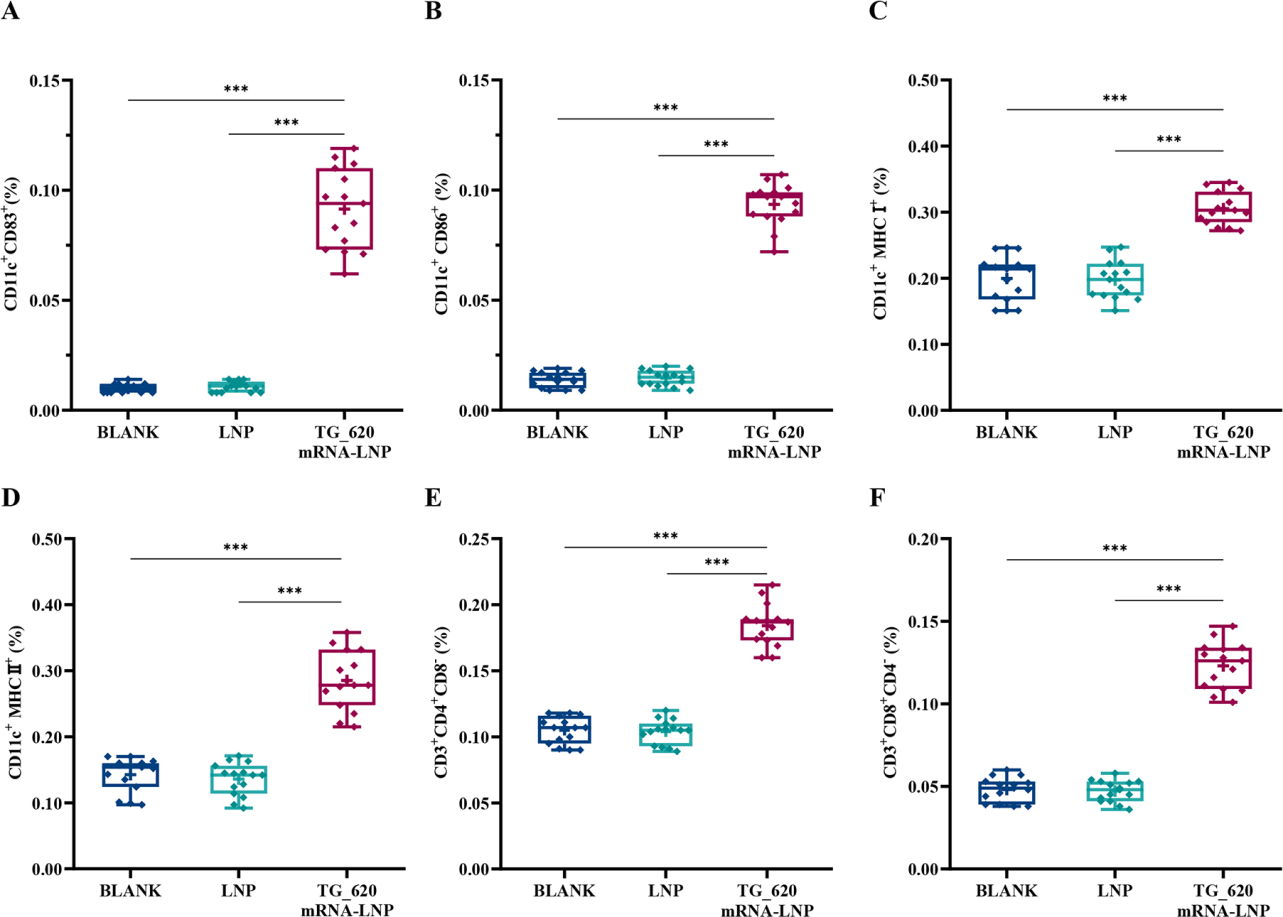
Flow cytometry analysis of DCs and T lymphocytes in immunized BALB/c mice. (**A**) Box graph showing the percentages of CD11c^+^ CD83^+^ DCs in mice splenocytes. (**B**) Box graph showing the percentages of CD11c^+^ CD86^+^ DCs in mice splenocytes. (**C**) Box graph showing the percentages of CD11c^+^ MHC-I^+^ DCs in mice splenocytes. (**D**) Box graph showing the percentages of CD11c^+^ MHC-II^+^ DCs in mice splenocytes. (**E**) Box graph showing the percentages of CD3^+^ CD4^+^ T lymphocytes. (**F**) Box graph showing the percentages of CD3^+^ CD8^+^ T lymphocytes. *n* = 5/group, ***P < 0.001. The data are shown as the means ± SD of three independent experiments.

Dendritic cells (DCs) can produce type I cytokines and IFN, which play an important role in innate immunity. In addition, DCs are the most potent antigen-presenting cells and can deliver antigenic information to T cells and activate them. Flow cytometry was used in this experiment to analyze CD83, CD86, MHC-II, and MHC-I levels to assess DC differentiation and antigen presentation. The CD4^+^ T and CD8^+^ T cells were measured using flow cytometry to analyze the specific immune response. As shown in Fig. 5A and B, the levels of CD83 and CD86 were significantly higher in TG_620 mRNA-LNP-immunized mice than in the BLANK and LNP groups. As shown in Fig. 5C and D, MHC-I and MHC-II levels were significantly higher in the TG_620 mRNA-LNP-immunized mice than in the BLANK and LNP groups. These results indicated that DCs were effectively activated and differentiated to present antigens. As shown in Fig. 5E and F, CD8^+^ and CD4^+^ T-cell levels were significantly higher in the TG_620 mRNA-LNP-immunized mice than in the BLANK and LNP groups. There were no significant differences between the control groups (*P* > 0.05). The results indicated that TG_620 mRNA-LNP effectively activated specific and efficient cell-mediated immunity, significantly killing intracellular parasites. These results are consistent with those of the CTL experiment and indicate the activation of specific and efficient cell-mediated immunity by TG_620 mRNA-LNP, which has great potential for restricting intracellular parasites.

### Cytokine-related transcription factor expression was increased after TG_620 mRNA-LNP immunization

We investigated key molecular pathways involved in TG_620 mRNA-LNP-induced immune protection. Interferon regulatory factor (*IRF*) expression can activate DCs to produce cytokines and IFNs, crucial in inducing specific cellular immunity. Nuclear factor kappa B (NF-κB) transcription factors are important in limiting *T. gondii* infection, and P65 is a critical component of the NF-κB family. Th1 cells are essential in restricting intracellular pathogen infections, and T-Box 21 (T-BET) can specifically promote Th0 differentiation into Th1 cells.

**Fig 6:**
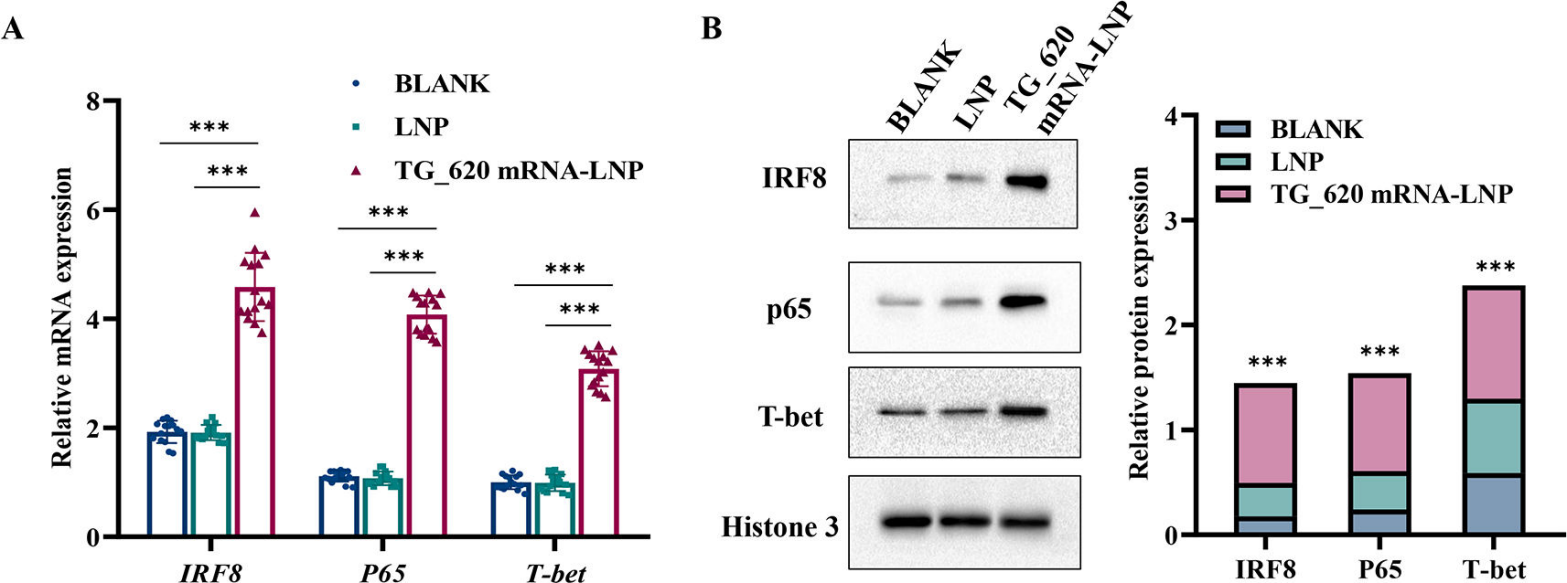
The mRNA and protein expression levels of IRF8, T-BET, and P65. (**A**) mRNA levels of *Irf8*, *Tbet*, and *p65*. *n* = 15/group, ***P < 0.001. (**B**) Protein levels of IRF8, T-BET, and P65. BLANK: splenocytes from untreated mice; LNP: splenocytes from the LNP-injected mice; mRNA-LNP: splenocytes from mice injected with TG_620 mRNA-LNP, ***P < 0.001.

As shown in Fig. 6A, the quantitative real-time reverse transcription PCR (qRT-PCR) results indicated that the mRNA levels of *Irf8*, *p65*, and *Tbet* were significantly upregulated in splenocytes from TG_620 mRNA-LNP-immunized mice compared with those in the LNP group and the BLANK control groups. There was no significant difference between the LNP and BLANK control groups. Additionally, as shown in Fig. 6B, the WB results indicated that the protein levels of IRF8, P65, and T-BET were significantly increased in splenocytes from TG_620 mRNA-LNP-immunized mice. There was no significant difference in protein levels between the LNP and BLANK control groups. The expression of the internal control gene (histone 3) was consistent among the three groups of samples. The WB and qRT-PCR results suggest that TG_620 mRNA-LNP immunization can effectively activate DC cells and the NF-κB pathway to induce an immune response. Furthermore, it can effectively stimulate Th1 cells and promote specific cellular immune responses.

### The survival time of immunized mice was prolonged after being challenged with *T. gondii*

**Fig 7:**
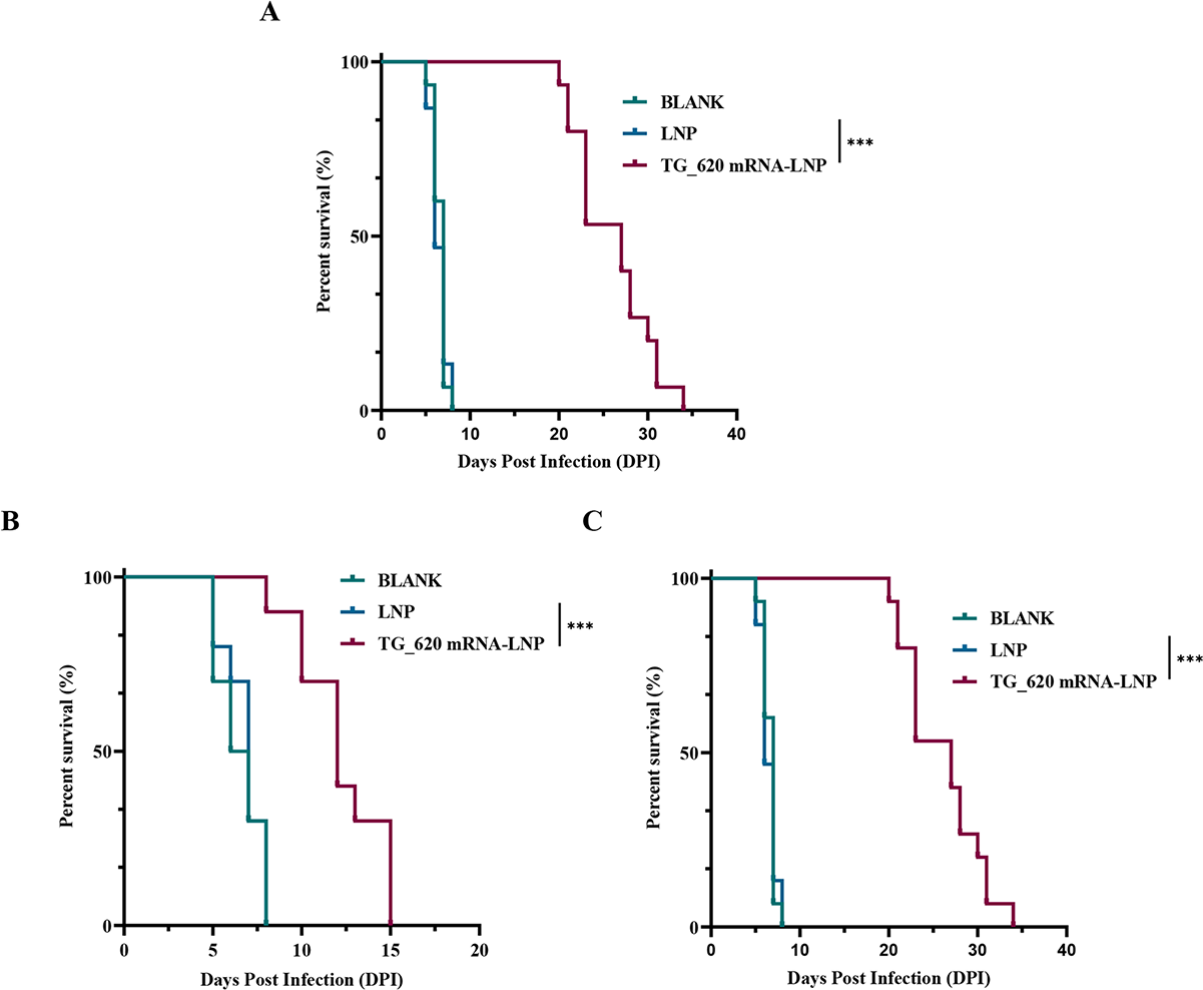
The survival rate of mice immunized with TG_620 mRNA-LNP or treated with adoptively transferred serum or splenocytes from vaccinated mice. (**A**) The survival rate of BALB/c mice immunized with TG_620 mRNA-LNP challenged with 1 × 10^2^ RH tachyzoites 2 weeks after the last immunization, *n* = 15/group. (**B**) The survival rate of mice receiving adoptively transferred serum (daily for 5 days) from mice vaccinated with TG_200 mRNA-LNP, LNP, or BLANK and challenged with 1 × 10^2^ RH tachyzoites, *n* = 10/group. (**C**) The survival rate of mice received adoptively transferred splenocytes (5 × 10^7^) from mice vaccinated with TG_620 mRNA-LNP, LNP, or BLANK and challenged with 1 × 10^2^ RH tachyzoites, *n* = 10/group.

The challenge with a lethal dose of *T. gondii* RH is the most direct way to evaluate the TG_620 mRNA-LNP-induced immune protection. Three groups of immunized mice were challenged with 1 × 10^2^ *T. gondii* RH strain, and their survival status was observed daily. As shown in Fig. 7A, the survival time of TG_620 mRNA-LNP-immunized mice was significantly prolonged after the *T. gondii* challenge (26 ± 3.43 days, *P* < 0.001), compared with that in the BLANK and LNP groups.

As shown in Fig. 7B, mice receiving serum from TG_620 mRNA-LNP-immunized mice had significantly prolonged survival time after being challenged with 1 × 10^2^ *T. gondii* RH strain (12.20 ± 2.40 days, *P* < 0.001), compared with that in the BLANK and LNP groups. As shown in Fig. 7C, mice receiving splenocytes from TG_620 mRNA-LNP-immunized mice had significantly prolonged survival time after being challenged with 1 × 10^2^ *T. gondii* RH strain (14.90 ± 2.85 days, *P* < 0.001), compared with that in the BLANK and LNP groups.

The results indicated that the TG_620 mRNA-LNP vaccine provides significant immune protection against *T. gondii* infection. Although adoptive transfer studies showed prolonged survival time in mice receiving TG_620-immunized mouse serum and splenocytes, the immune protection was limited.

## DISCUSSION

Toxoplasmosis poses a significant public health concern and seriously threatens economic and social productivity. Although numerous anti-*T. gondii* vaccines have been studied for their immunogenic efficacy in defending against acute toxoplasmosis, few vaccines offer complete protection, and safety and efficacy issues persist (9) (19). Therefore, a safe and effective anti-*T. gondii* vaccine is urgently needed. Among the various types of vaccines studied, mRNA vaccines hold great promise to prevent epidemics of infectious diseases because of their potential for high potency, rapid development, safe administration, and low-cost manufacture (20).

Secretory proteins play an important role in host-pathogen interactions; therefore, the search for vaccine candidate molecules on the ToxoDB focused on predicting *T. gondii* secretory proteins. Bioinformatic databases can offer a wide range of targets for vaccine development in pathogen vaccine research. Various methods, such as Vaxijen and AntigenPro servers, have been employed to predict the antigenicity of candidate vaccines (21 – 24). After screening for genome-wide secreted proteins containing transmembrane structural domains and signal peptides, further T-/B-cell epitope prediction of the screened candidate molecules revealed that the TG_620 molecule had the best-predicted scores of T-/B-cell epitopes, suggesting that TG_620 has great potential as a vaccine candidate. Furthermore, the percent IC_50_ value of the TG_620 peptide was lower than that of SAG1, and the linear B-cell epitope score of TG_620 peptide was higher, indicating that the T-cell epitopes of TG_620 are predicted to have strong binding to MHC-II molecules. Further prediction suggested that TG_620 has significant potential for inducing a specific cellular immune response, making it a promising candidate for vaccine production.

B-cell-mediated humoral immune response protects against *T. gondii* infection (25). Mice lacking B cells experience earlier mortality during the chronic stage of infection (26). Our results showed that levels of IgG and its subtypes (IgG2a and IgG1) increased significantly, and a mixed Th1 and Th2 immune response with a Th1 bias was induced after TG_620 mRNA-LNP immunization. The Th1-type immune response is critical in effectively resisting *T. gondii* infection under natural conditions (27). Numerous studies have suggested an important role for IgG antibody production in the fight against *T. gondii* infection (28 – 30).

A high level of Th1 cytokine immune response plays a critical role in host defense against *T. gondii* infection, with IFN-γ and IL-12 being the primary mediators of the Th1 immune response. IFN-γ is secreted by natural killer (NK) cells through a toll-like receptor myeloid differentiation factor 88-dependent signaling pathway synergistically triggered by *T. gondii* antigen and IL-12 (31, 32). IL-12p35/p40^−/−^ mice exhibited a significant reduction in IFN-γ levels, while IL-12^−/−^ mice showed diminished control over parasites and an increased parasite burden at the site of infection (33, 34). IL-12 produced by DCs and macrophages triggers the proliferation of NK cells, CD4^+^ T cells, and CD8^+^ T cells, thus mediating cytotoxicity and the production of large amounts of IFN-γ (35). The TG_620 mRNA-LNP could produce IFN-γ and IL-12, indicating that it could induce an effective immune response against *T. gondii*. However, our study also observed elevated levels of the anti-inflammatory cytokines IL-4 and IL-10. IL-10 plays an important role *in vivo* and can downregulate the response of single factors and IFN-γ to acute intracellular infection, thereby preventing host immune pathology. Studies have shown that the death of IL-10^−/−^ mice is associated with liver pathology and severe immune pathology mediated by Th1 immune responses in the intestine (36, 37). By contrast, IL-4 is protective against the development of toxoplasma encephalitis by preventing the formation of *T. gondii* cysts and the proliferation of tachyzoites in the brain (38). The TG_620 mRNA-LNP has a suppressive effect on immune pathology. Some previous *T. gondii* vaccines provide only Th1 immune responses without inducing Th2 immunity (39 – 41). The cytokine detection results were consistent with the antibody subtype detection results, indicating that TG_620 mRNA-LNP can induce both Th1 and Th2 immune responses in mice, with a predominance of the Th1 immune response.

The lymphocyte proliferation test is a classical test to detect cellular immune function. The results showed that TG_620 mRNA-LNP induced specific cellular immunity. CTLs with *T. gondii* antigenic specificity are vital in controlling *T. gondii* replication. A single CTL can kill multiple target cells continuously, and the killing efficiency is high. CTL experiments can be conducted *in vivo* or *in vitro* (42). However, most studies about *T. gondii* vaccines did not involve CTL evaluations; only a few evaluated it *in vitro* (40, 41, 43, 44). Specific activation of T lymphocytes might play an important role in developing spleen lymphocytes in TG_620 mRNA-LNP-immunized mice and induce a *T. gondii*-specific cellular immune response.

After infection with the parasite, the expression levels of CD83, CD86, MHC-I, and MHC-II markers on splenic DCs increased (45). DCs, which produce type I cytokines and interferon, play a role in innate immunity, function in antigen presentation, and mediate T-cell killing of intracellular pathogens (46). When the CD86 and CD28 molecules bind, they provide co-stimulatory signals from T cells, lowering the initial activation threshold (47). CD83 is often expressed on the surface of mature DCs, which plays an important role in immune regulation and inflammation resolution (48). Our results indicated that DCs were activated and mature and could enhance MHC-I and MHC-II molecules to present antigens. Activated CD4^+^ T lymphocytes can activate macrophages, producing cytokines recruited to the site of infection (49). Activated CD8^+^ T cells can differentiate into CTLs, which exhibit immunotoxicity against the parasite (49). During the acute phase of infection, host resistance mainly involves NK cells and CD4^+^ T cells, while in chronically infected mice, the main cells producing IFN-γ to control the parasite are CD8^+^ T cells, with a small proportion of CD4^+^ T cells (50). Our results showed that T cells were also effectively activated. Overall, the TG_620 mRNA-LNP immunization can promote (i) the binding of MHC/peptide complexes to TCR, (ii) ligand-triggered co-stimulatory receptor signaling on antigen-presenting cells, and (iii) integration of the cytokine milieu for T-cell differentiation, thereby facilitating the three regulated signals essential for *T. gondii* antigen presentation, T-cell response, and cell activation (51). Similar studies have been conducted; however, most focused on CD8^+^ T and CD4^+^ T cells (41, 43, 52) without examining DC cells.

IRF8 is considered essential for developing plasmacytoid DCs and type I conventional DCs, and it maintains high expression in differentiated DCs (53). Besides, it is crucial for host resistance against *T. gondii*, and its deficiency can lead to acute susceptibility of mice to the parasite (53). T-BET is a key factor for the terminal maturation of NK cells and maintenance of peripheral homeostasis and is likewise required for the Th1 response (54). The T-BET-dependent mechanism is crucial for survival during the acute phase of infection: mice deficient in T-BET die faster from *T. gondii* infection than mice lacking T cells (54). NF-κB is a transcription factor and enhancer of activated B cells. It plays a key role in blocking *T. gondii*-mediated apoptosis, and this inhibition is lost in p65^−/−^ cells (55). Our results suggest that the increase in IFN-γ production induced by TG_620 mRNA-LNP vaccination might be attributed to the activation of the IRF8 and NF-κB pathways and the activation of CD4^+^ T cells and NK cells mediated by T-BET. Several previous studies have also suggested that IFN-γ production is mediated by the activation of the IRF8, T-BET, and NF-κB pathways via this mechanism (41, 43, 56 – 58).

Survival time is a direct and reliable indicator of the safety and efficacy of *T. gondii* candidate vaccines. Although the TG_620 mRNA-LNP vaccine did not induce complete protection against high virulence *T. gondii* infection, it did induce better partial protection than other *T. gondii* vaccines, such that the survival time of the mice in this study was longer than that in some previous studies (43, 59) and even longer than that of the combined DNA vaccine (60). Mice immunized with TG_620 mRNA-LNP were able to elicit both humoral and cellular immune responses against *T. gondii*. We used adoptive transfer to investigate whether the serum and splenocytes from mice immunized with TG_620 mRNA-LNP had sufficient inhibitory effects on the parasite. Following the challenge with *T. gondii* RH, it was found that the survival time of naive mice receiving serum and splenocytes from mice immunized with TG_620 mRNA-LNP was significantly prolonged. Only a few previous *T. gondii* vaccine studies have evaluated adoptive transfer (61). The transfer of splenocytes is more involved than the transfer of serum in *T. gondii* vaccine studies (62, 63). In addition, splenocyte transfer experiments have been used in research into other parasites, such as *Trypanosoma cruzi* and *Leishmania donovani* (64, 65).

In summary, the TG_620 mRNA-LNP vaccine induces a specific immune response and can effectively protect BALB/C mice against *T. gondii* infection, representing a promising candidate for the *T. gondii* vaccine. To better prevent *T. gondii* infection, selecting appropriate adjuvants or combining the vaccine with other important *T. gondii* molecules might be necessary. Previous studies have shown that the protective effect of multiple proteins or epitopes is superior to that of a single protein or epitope (8, 66). Constructing a multi-epitope vaccine comprising TG_620 combined with other promising known or hypothesized *T. gondii* molecules and adjuvants might provide better anti-*T. gondii* immune protection.

## MATERIALS AND METHODS

### Epitope prediction

In this study, the DNA sequence of *T. gondii* TG_620 was obtained from the database ToxoDB (http://toxodb.org/toxo/). The IEDB (http://tools.immuneepitope.org/mhcii/) online analysis system and DNASTAR software (Madison, WI, USA) were used to analyze the coding region of the gene sequence. Protean software in DNASTAR was used to predict hydrophilicity flexible regions and calculate the antigenic index and surface probability. The IEDB online analysis system was used to measure antibody sensitivity and analyze semi-maximum inhibitory concentration (IC_50_) values of peptides bound to TG_620 MHC class II molecules.

### Animals

Specific pathogen-free female BALB/c mice aged 6–8 weeks and New Zealand white rabbits aged 7–9 weeks were obtained from the Zhejiang Experimental Animal Center (Hangzhou, China). All mice used for the experiments were raised and handled strictly following the Good Animal Practice requirements of the Animal Ethics Procedures and Guidelines of the People’s Republic of China. The procedures in this study involving mice and rabbits followed Chinese legislation on using and caring for laboratory animals (GB/T35823-2018). The animal experiments were also approved by the Hangzhou Medical College Institutional Animal Care and Use Committee (No: 2021-152). Mice were euthanized by intraperitoneal injection of sodium pentobarbital (150 mg/kg).

### Parasites and cells

*T. gondii* RH strain (genotype I) was propagated and harvested in human foreskin fibroblast (HFF) cells. Human embryonic kidney 293-T cells (293T cells) and mouse muscle cells (C2C12 cells) were used for the transfection experiments. The lipo2000 reagent (Invitrogen, Carlsbad, CA, USA) was used for the transfection experiments. HFF, 293T, and C2C12 cells were purchased from the American Type Culture Collection Manassas, VA, USA) and maintained in Dulbecco’s modified Eagle’s medium containing 10% fetal bovine serum (Gibco, Auckland, New Zealand) in an incubator with 5% CO_2_ at 37°C.

### Preparation of rabbit anti-TG_620 polyclonal antibodies

The TRIzol reagent (Invitrogen) was used to isolate total RNA from 1 × 10^8^ tachyzoites of *T. gondii*. A GoScript Reverse Transcription System (Promega, Madison, WI, USA) was then used to synthesize the cDNA, followed by PCR to amplify the complete TG_620 gene of *T. gondii*. DNASTAR software was used to design primers (forward: 5′-CGGAATTCATGATACGGTTCTTTCAGGTCGCTT-3′; reverse: 5′-CCAAGCTTTCATAGCCCTTCAGAGCACAC-3′). The PCR product was ligated into the pET28a vector via restriction endonuclease sites (EcoRI /HindIII) and identified by sequencing. The plasmid was named as pET28a-TG_620. The recombinant TG_620 protein (rTG_620) was expressed in *Escherichia coli* BL21 (DE3) overnight at 37°C *in vitro* using 0.1-mM/L isopropyl-beta-D-thiogalactoside. The bacteria were centrifuged at 12,000  ×  *g*, 4°C for 15  min and resuspended with phosphate-buffered saline (PBS). Cells were cracked by ultrasound at low temperature (power: 200 W, 5-s ultrasonic interval 10 s, 100 times), and supernatant was collected. Ni^2+^-NTA agarose columns (Qiagen, Hilden, Germany) were used to purify rTG_620 using affinity chromatography. The purified rTG_620 was utilized to generate rabbit anti-TG_620 polyclonal antibodies as described by Zheng et al. (43).

A rabbit was immunized subcutaneously with purified rTG_620 protein (200 µg) three times, with an interval of 2 weeks between each immunization. Blood was collected from the ear vein of the rabbits 2 weeks after each injection, and the serum containing the anti-rTG_620 polyclonal antibodies was separated and stored at −80°C.

### Generation of modified mRNA and *in vitro* transcription

**Fig 8:**
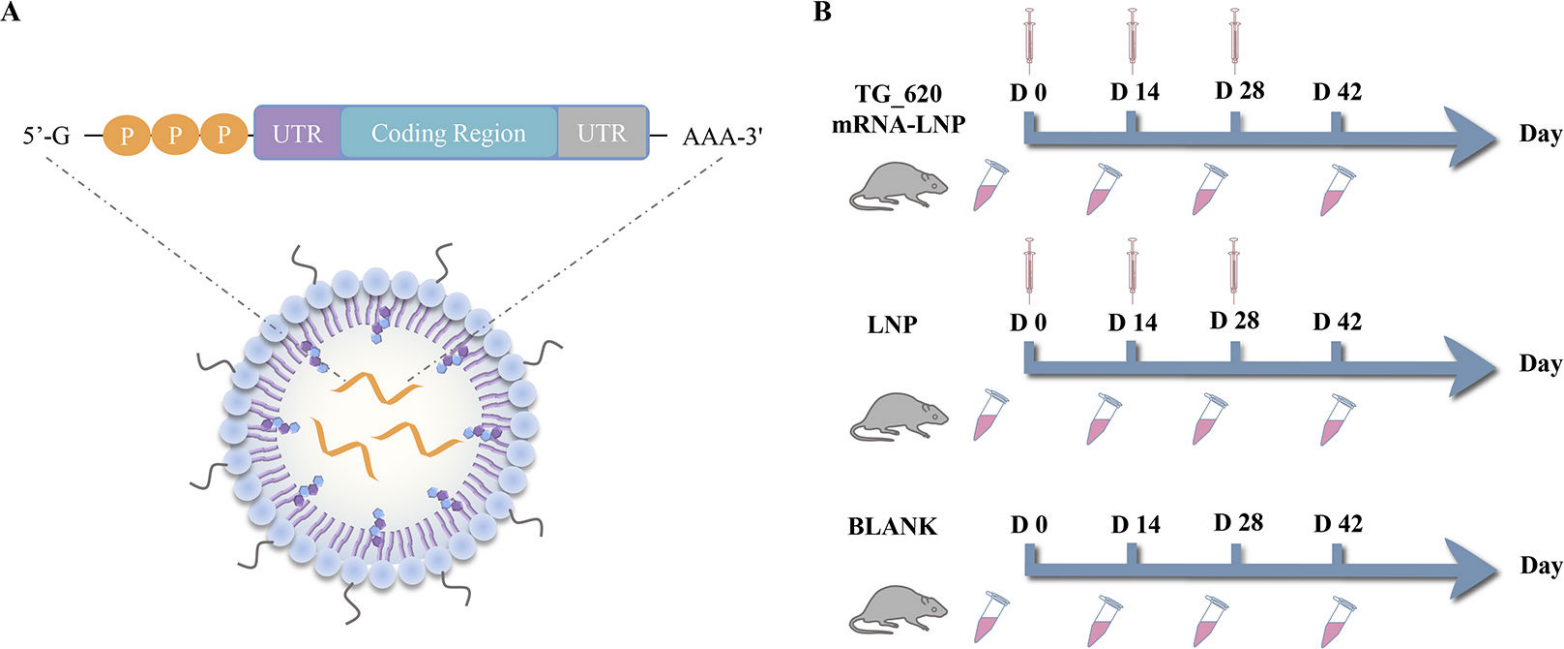
Experimental design and immunization schematic diagram. (**A**) Schematic diagram of the construction of TG_620 mRNA-LNP vaccine candidates and a schematic diagram showing the proposed mechanism for mRNA vaccine candidate translation in the cytoplasm. (**B**) Scheme of the TG_620 mRNA-LNP vaccination schedule.

The coding region of the TG_620 was flanked by the optimized 5′ and 3′ untranslated regions (UTRs), a poly A tail, and a 5′ cap structure (Fig. 8A; Fig. S1). The 500-bp sequences, chosen as the 5′ and 3′ UTRs via ToxoDB analysis, were placed before and after the TG_620 coding sequence of the mRNA construct. The 5′ cap and poly-A tail structures are added enzymatically. The modified mRNA was designed and synthesized from linearized DNA under the control of a T7 promoter using a T7-FlashScribe Transcription Kit (CellScript, Madison, WI, USA). When performing *in vitro* transcription, the standard unmodified nucleotides were used as described by the manufacturer. When generating mRNA for encapsulation in LNPs, uridine was replaced by pseudo-uridine using the Incognito mRNA Synthesis Kit (CellScript). The constructed mRNA was stored at −80°C until use.

### Generation of mRNA/LNP and the EE

mRNA-LNPs were prepared using GenVoy ionizable lipid mixture (GenVoy-ILM) on the benchtop (PNI, Santa Rosa, CA, USA). A total flow rate of 12 mL/min was set such that mRNA and GenVoy-ILM flow ratios of 3:1 were used to generate the mRNA-LNPs in PNI buffer through laminar flow tubes coated with lipids. GenVoy-ILM was a mixture of molar ratios 50 (PNI ionizable lipids):10 (distearoylphosphatidylcholine): 37.5 (cholesterol): 2.5 (PNI stabilizer). To determine the EE, the percentages of encapsulated RNA were determined by detecting the RNA concentrations both outside and inside the LNPs using Quant-it RiboGreen RNA Reagent and Kit (Thermo Fisher Scientific, Waltham, MA, USA) according to the manufacturer’s instructions. Finally, the EE was calculated according to the following formula (where *W* represents weight): EE% = *W* (total RNA) − *W* (free RNA)/*W* (total RNA). The encapsulated mRNA was stored at 4°C for extended periods.

### Immunization and *T. gondii* challenge

Mice were randomly divided into three groups of 20. Of the 20 mice in each group, 15 were used for immunological studies to test antibody levels, and five were used to extract splenocytes and serum for follow-up immunological experiments and adoptive transfer studies. Twenty mice were vaccinated with TG_620 mRNA-LNP at 100 µL (1 mg/mL) three times intramuscularly as the experimental group. Twenty mice were injected with LNP 100 µL as the negative control group, and the other 20 were the BLANK control group. The immunization schedule is shown in Fig. 8B. At 2 weeks after the final immunization, each group of mice was subjected to a challenge experiment using 1 × 10^2^ tachyzoites of *T. gondii* RH strain. The status of each group of mice was observed daily, and survival time was recorded.

### ELISA analysis of the levels of antibodies and cytokines

Serum was collected from mice before each immunization and 2 weeks after the third immunization to test for antibodies. The total serum IgG and subtype antibodies (IgG1 and IgG2a) were detected indirectly by an ELISA. 96-well plates were coated with 10-µg/mL rTG_620 protein. The detailed assay procedures are in our previous publication (43). Splenocyte IL-4 levels at 24 h, IL-10 levels at 72 h, and IFN-γ and IL-12 levels at 96 h were measured using commercially available kits (eBioscience, San Diego, CA, USA) according to the manufacturer’s instructions. Three replicates were included for each sample.

### Lymphocyte proliferation assay and CTL activity assays

As in our previous study (43), after erythrocytes were lysed using red blood cell lysis buffer (Sigma, St. Louis, MO, USA), the splenocytes were cultured in triplicate in 96-well plates (2 × 10^5^ cells/well) in the presence of 10-µg/mL rTG_620 protein. According to the manufacturer’s protocol, the proliferative activity of spleen lymphocytes was determined using the Cell Counting Kit 8 assay (Dojindo, Kumamoto, Japan). The stimulation index (SI) was then calculated using the following formula: SI = (OD_570_ rTG_620/OD_570_ Control): (OD_570_ ConA/OD_570_ Control).

The CytoTox 96 Non-Radioactive Cytotoxicity Assay Kit (Promega, Madison, WI, USA) was used to detect CTL activity. Mouse splenocytes were cultured as effector cells with 100-U/mL recombinant IL-12 for 5 days. The target cells were Sp2/0 mice cells transfected with TG_620 mRNA-LNP. Effector cells were mixed with target cells at ratios of 80:1, 40:1, 20:1, and 10:1. The cytotoxicity was measured according to the manufacturer’s instructions.

### WB analysis and quantitative real-time PCR

T-BET, P65, and IRF8 antibodies were purchased from Cell Signaling Technology, Inc. (Danvers, MA, USA). WB detects intranuclear transcription factors (NF-κB p65, T-BET, and IRF8) using a nuclear and cytoplasmic isolation kit (Beyotime Institute, Haimen, China). Horseradish peroxidase-conjugated goat anti-mouse T-BET, P65, and IRF8 diluted to 1:2,000 were used to detect the bound antibodies. Detailed assay procedures can be found in our previous study (43).

qPCR experiments were performed using a CFX96 connect apparatus (Bio-Rad, Hercules, CA, USA) and cDNA prepared by reverse transcription of isolated RNA as the template. Three replicate reactions were performed using the SYBR Green-based GoTaq qPCR Master Mix (Promega) according to the manufacturer’s instructions. The primers used are listed in Table 3.

**TABLE 3:** IC_50_ values for TG_620 and SAG1 binding to MHC class II molecules were obtained using the IEDB^*a*^

MHC-II allele^*b*^	Start-stop^*c*^		Percentile rank^*d*^	
	SAG1	TG_620	SAG1	TG_620
H2-IAb	26–40	3–17	0.95	0.33
H2-IAd	21–35	8–22	2.85	0.04
H2-IEd	14–28	42–56	3.35	3.95
HLA-DRB1*01:01	12–26	2–16	1.80	0.01

^*a*^ The Immune Epitope Database (http://tools.immuneepitope.org/mhcii).

^*b*^ H2-IAb, H2-IAd, and H2-IEd alleles are mouse MHC class II molecules; the HLA-DRB1*01:01 allele is a human MHC class II molecule.

^*c*^ Fifteen amino acids were chosen for analysis.

^*d*^ Low percentile indicates high-level binding according to the software instructions.

### Flow cytometry

Splenocytes were resuspended in 100 µL of PBS, and cells were stained with anti-mouse CD11c-fluorescein isothiocyanate (FITC), CD83-phycoerythrin (PE), CD86-PE, MHC-I-PE, MHC-II-PE, CD3e-FITC, CD4-PE, CD3e-PE, and CD8-PE (eBioscience, San Diego, CA, USA) for 40 min at 4°C in the dark. A flow cytometer (Beckman Coulter Inc., Brea, CA, USA) was used for cell fluorescence analysis. All samples were stained using the above strategy and subjected to flow cytometric analysis. All labeled cells were analyzed on a FACS Aria III flow cytometer (BD Biosciences), and data were analyzed using CellQuest software (BD Biosciences, Franklin Lakes, NJ, USA).

### Adoptive transfer study

Serum adoptive transfer study. Mice were randomly divided into three groups of 10 mice each. Each group of mice received 100 µL of serum from TG_620 mRNA-LNP-immunized mice, LNP-immunized mice, and naive mice, respectively. Five days after continuous adoptive transfer, the mice were challenged with 1 × 10^2^ RH tachyzoites, and their survival was monitored daily. In addition, similarly constituted groups of mice received 5 × 10^7^ splenocytes from TG_620 mRNA-LNP-immunized mice, LNP-immunized mice, and naive mice, respectively. After 24 h, each mice group was challenged with 1 × 10^2^ RH tachyzoites per mouse. The status of each group of mice was observed daily, and survival was recorded.

### Statistical analysis

GraphPad 8.0 software (GraphPad Prism, San Diego, CA, USA) was used for one-way analysis of variance (ANOVA) and log-rank tests. One-way ANOVA was used for antibody, cytokine, lymphocyte proliferation levels, CTLs, and flow cytometry data analysis. The log-rank test was used for survival analysis. Differences were considered statistically significant when *P* < 0.05.

## Data Availability

The data sets generated and/or analyzed during the current study are available from the corresponding author upon reasonable request.

## SUPPLEMENTAL MATERIAL

Supplemental file S1 spectrum.02866-23-s0001.docx Presentation of TGGT1_278620 construct sequences.

ASM does not own the copyrights to Supplemental Material that may be linked to, or accessed through, an article. The authors have granted ASM a non-exclusive, world-wide license to publish the Supplemental Material files. Please contact the corresponding author directly for reuse.
