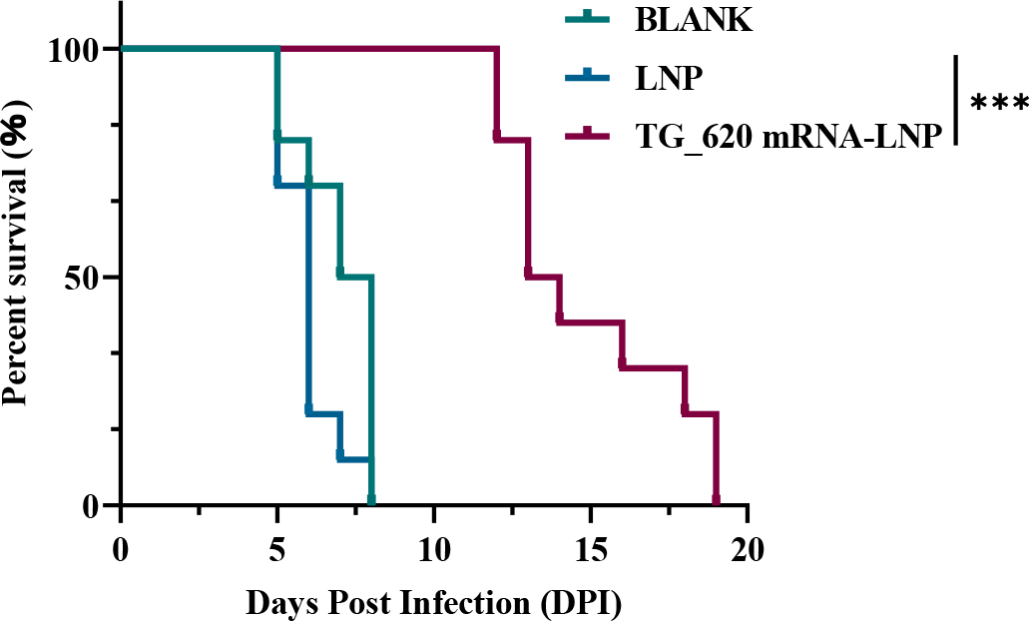# Correction for Zhang et al., “A novel mRNA vaccine, TGGT1_278620 mRNA-LNP, prolongs the survival time in BALB/c mice with acute toxoplasmosis”

**DOI:** 10.1128/spectrum.00596-24

**Published:** 2024-05-07

**Authors:** Yizhuo Zhang, Shiyu Li, Hongkun Chu, Jing Li, Shaohong Lu, Bin Zheng

## AUTHOR CORRECTION

Volume 12, no. 1, e02866-23, 2024, https://doi.org/10.1128/spectrum.02866-23. Page 4: Table 1, which lists qRT-PCR primers, should be Table 3.

Page 9: Figure 7C should appear as shown in this correction. The original panel was an inadvertent duplicate of Fig. 7A.

Page 14: Table 3, which lists IC_50_ values, should be Table 1.

We apologize for these errors, which did not change the final results.

**Fig 7:**