# Supplementary material for: A novel mRNA vaccine, TGGT1_278620 mRNA-LNP, prolongs the survival time in BALB/c mice with acute toxoplasmosis
Source: Microbiol Spectr. 2023 Dec 1;12(1):e02866-23. doi: 10.1128/spectrum.02866-23 (PMC10783036; doi:10.1128/spectrum.02866-23)
Supplement: Supplemental file S1 — Presentation of TGGT1_278620 construct sequences. [file spectrum.02866-23-s0001.docx]

**AATAATACGACTCACTATAGGGG**CACTTCCTTTTTGGATCGATAACATCCAGATAACCATGACAACAACGCCGTGTTTCGTAAAAAAGCTGAAGTCCAGTCTCATTTGTACCAAAAAGTTGACTCATCTTACACCGCAGCACCTGATTTTGCCAATGACAACAGTCATTTTCGGCAGCTGTTTTCAGACGTGAGTGCTTTGTAGACACGATCGTTGCTGTGACGCATCCCTCTCAGCTCAGACTTTCCATTTCTACACGAATCCCTCATCACTGGGAAGCAAAGGAACATCTGGTCCCAGTGAGCCTGGGGATGAGTCGTCCTCTGAGTAAGCCTACGTGTCTCTGTACGTTTCACCAAGCAGTCAAACACGGCAGCGTCTGGTGCGTCACAGGTAGGTCACCTGTATGTACACCGGATGCAACGGTCCCCCGACAAAACACTGGATATGTATGGTTTGAGAGTGTTGCTGCACAGTTCTCGGGGTAGAAAACGGAAGTACAGTGGAGCACTGCAGGACTGCAAATGATACGGTTCTTTCAGGTCGCTTTCGCGGCCTCCTCTATGCTGGCGTCTCTTGCCAGTGCACGTTTTGACTTCGAAAATGTGCATTCTCATCAGACACAAGGCCTGATTAGTGGAACAGGCTCTCCCGTTGTCAGTGTCGTCCGTCGGCCGGGAGGCAGACGAGTATGGAAAATTGAGGCAGAGGCTGAACAAAGACTGCTTGAGAAGGAACTCGGCAAGATGACGGCACTTGCCAGGGAGATGCAAACGGTATTAAATACTGCACAGTGGTCACCGCCAGAGTGGCAAGGACTTAACATAGCTGTACTAATGAGAAACCAGCGAGATTTCGAAAAAGCGCTCAAAGACATTGCGGAAGCCGCGTCTGAGGAAAAGAGGAGAATAATTAGAGACTTGGCAGCAAGCAACATCGCCGACTGCCCAGAATGCCGTGCTATTCCACCCCGTGAATCGGGCGGCGCTTCTGAAGAGGAAGCATCAACCGTGGAGACTCCCACAGATGAGGTTCCTCCTGAAGAAGACGTGCCGTCTGTCCCAGACGATGAGGGAACAGAAGATGTGCCAGAACGGACGCCTGGTCCTGCCGAAGAGGTTCCTCCTGAAGAAGCTGAGCCCCCAGAGAATGAAGGGACAAAAGAAGCACCAGAGCAGAGACCTTCCACTGGCACGCAGGCTCCCCTTCCATCGTCTGAAGAGGTTCCTCCAGCCTCTGCTGATGAGGCTCCTGAAGAACCTACGGCGCAGGAGGAGATACCACAACCTACAGTCCCTGAGCCTTCGCCAATTAAGGGACCAGTAACGCCGGAGCTCACTCCACAGGAAAAGGTGTGCTCTGAAGGGCTATGAATAGTTCTATGGGGCAAACGCAAGTGTTCGACCTTTTTCCATGCAGGAAAGCCTCATGCAAAAGGCGGAGGCGGCAGAAAAGTTCCGACAGAACCCTTGGTGAGTCGACACTCATGGGAACCTATTTTGCTTGGATGTAAAAGCACACTGCCAGGCTATGGTTCTACCTGCTCGTGGTCCTGCCCTCTGCCATTACGGTGATTCTTCTTGCCCTTGCTATGTTCATTTAACAGACACTGTGCGCTACGTATCCGCATGGGAAACAATCATATGGTGACGATTAGTGGTGGCGTATGATTAATCATGGACAAAATGCTTCGATCCCAGCAACTATTAGCGAAGAAGATGCCGCGCCAACGAATCTGCATTGCAAACGCTACTTAGCGTTGTCTGTTCACCAAGTCGGAAAAGCCGGCAATTATACAGCTAGCAACGGAACTTGTATTAGGGAAACCCCATGCCGGAAGACGGCCGGCATGCTGTGTGCAGCGCTCATGCTGC

**Supplement 1.** The presentation of TGGT1_278620 construct sequences. The T7 promoter sequence is highlighted in yellow, the 5' UTR sequence is highlighted in blue, the TGGT1_278620 coding sequence is highlighted in red, and the 3' UTR sequence is displayed in gray font. We used the 500bp upstream and 300bp downstream sequences of the TGGT1_278620 gene for the 5' UTR and 3' UTR, respectively.
